# A Ganglion Cyst in the Anterior Cruciate Ligament of a 13-Year-Old Boy

**DOI:** 10.7759/cureus.37692

**Published:** 2023-04-17

**Authors:** Yousef S AlGhamdi, Ben Maitigue Mahmoud, Sultan M AlBlaui, Khaled K Altaraman, Khalid E Alhumam, Ibrahim A AlSinan, Abdullah M AlHossan

**Affiliations:** 1 Orthopedic Surgery, King Fahad Military Medical Complex, Dhahran, SAU; 2 Orthopedic Surgery, Sahloul University Hospital, Sousse, TUN; 3 Orthopedic Surgery, King Abdulaziz Hospital, Alahsa, SAU; 4 Orthopedic Surgery, Imam Abdulrahman Al Faisal Hospital, Dammam, SAU; 5 College of Medicine, Alfaisal University, Riyadh, SAU

**Keywords:** pediatric knee, arthroscopy knee, ganglion cyst aspiration, knee ganglion cyst, anterior cruciate ligament (acl)

## Abstract

Intra-articular ganglion cysts of the knee are a rare occurrence in pediatric patients, particularly involving the anterior cruciate ligament (ACL). Only a handful of case reports have been documented in the medical literature, highlighting the rarity of this condition. Patients with intra-articular cysts often experience knee discomfort and mechanical symptoms like locking of the knee.

We present the case of a 13-year-old boy who had a unilateral intra-articular ganglion cyst of the ACL in his left knee. To diagnose and treat the cyst, we conducted radiographs and MRIs, and arthroscopic drainage was performed, resulting in a successful cyst decompression.

Our case report provides an overview of the pathogenesis, diagnostic methods, treatment options, and complications of treatment for intra-articular cysts of the ACL. It highlights the rarity of this condition in pediatric patients and underscores the importance of prompt diagnosis and appropriate management.

## Introduction

Ganglion cysts are a rare occurrence within the joint, with the first recorded instance of an intra-articular cyst being reported by Caan in 1924 following a routine autopsy [1]. Since then, literature on the subject has grown due to the increasing availability of MRI and the expanding use of diagnostic arthroscopy. A diagnosis of a ganglion cyst based solely on clinical grounds is uncommon, and frequently, it is the MRI findings that confirm the diagnosis. In this case report, we present a ganglion cyst located within the Anterior Cruciate Ligament (ACL) of a pediatric patient who began experiencing symptoms after a traumatic event. Our objective is to highlight the diagnostic importance of MRI in identifying intra-articular ganglion cysts, particularly in the case of rare anatomical locations such as the ACL.

## Case presentation

A 13-year-old male without any known chronic medical conditions presented to our outpatient department with a history of left knee pain for over four years. According to the patient and his parents, the pain started after a fall from a swing. However, they denied any history of weight loss or other constitutional symptoms. Physical activity, particularly playing soccer, exacerbated the pain. Upon clinical examination, mild swelling of the left knee, was observed with no joint effusion. The patellar grinding test was positive, and the active range of motion was limited with a 10-degree loss of extension. Assessment of the ligaments under stress revealed a stable knee with a regular gait cycle.

**Figure 1:**
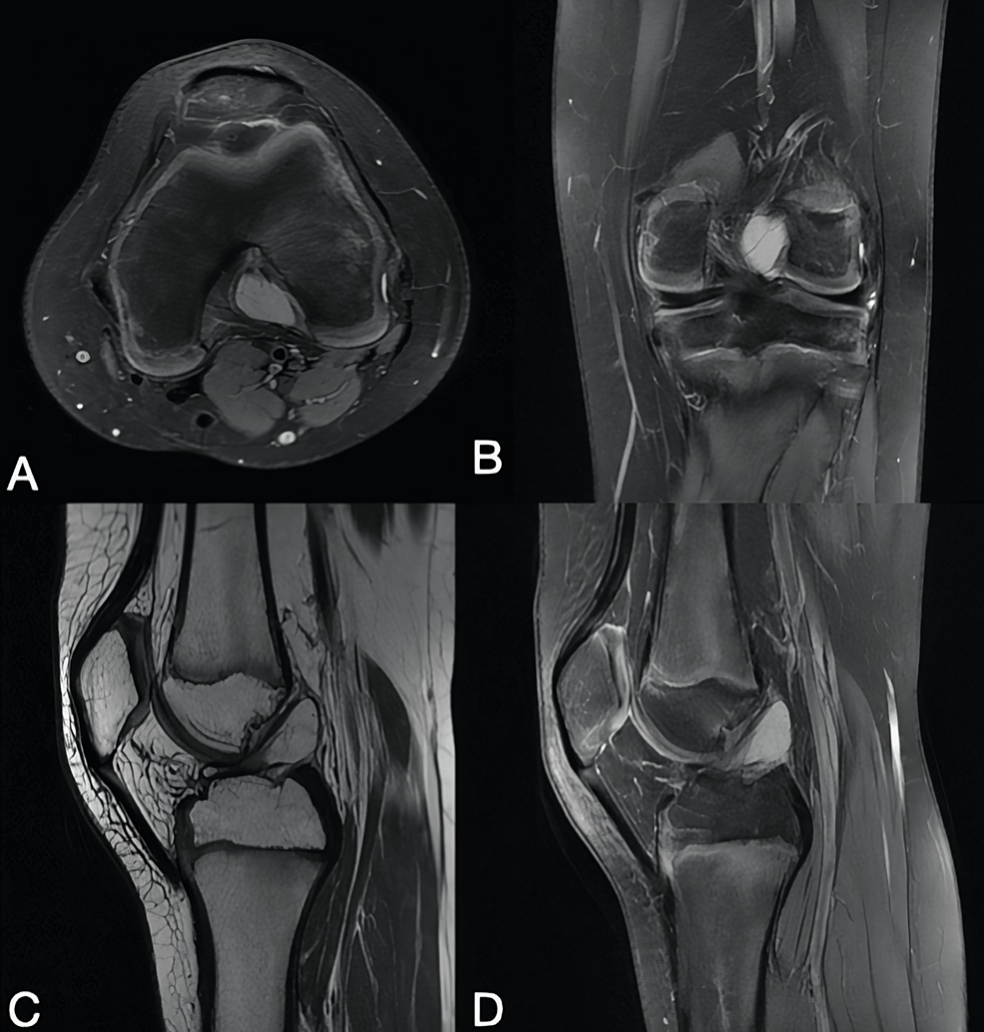
A: Preoperative left knee MRI in axial, B: Coronal, C and D: Sagittal planes " C, D". revealed a well-defined lesion with fluid signal intensity in the intercondylar notch, measuring 2.1 x1.7 x1.5 mm, which was eccentric to compression and closely related to the anterior cruciate ligament. A corticated bone fragment was noted within Hoffa's fat pad.

Initially, the patient was diagnosed with patellofemoral syndrome. However, after physiotherapy and nonsteroidal anti-inflammatory medications (NSAIDs) failed to provide relief, an MRI of the left knee was recommended (Figure 1).

**Figure 2:**
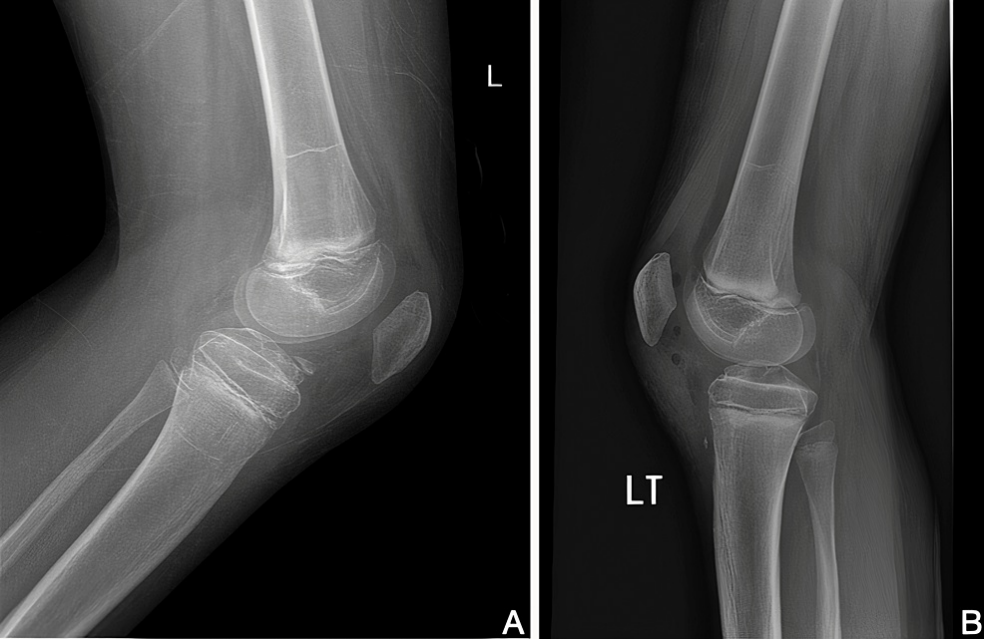
A: Showing a lateral X-ray of the left knee preoperatively reveals a partial bony avulsion of the tibial spine. B: Showing a lateral X-ray of the left knee postoperatively confirms the excision of the avulsed part.

**Figure 3:**
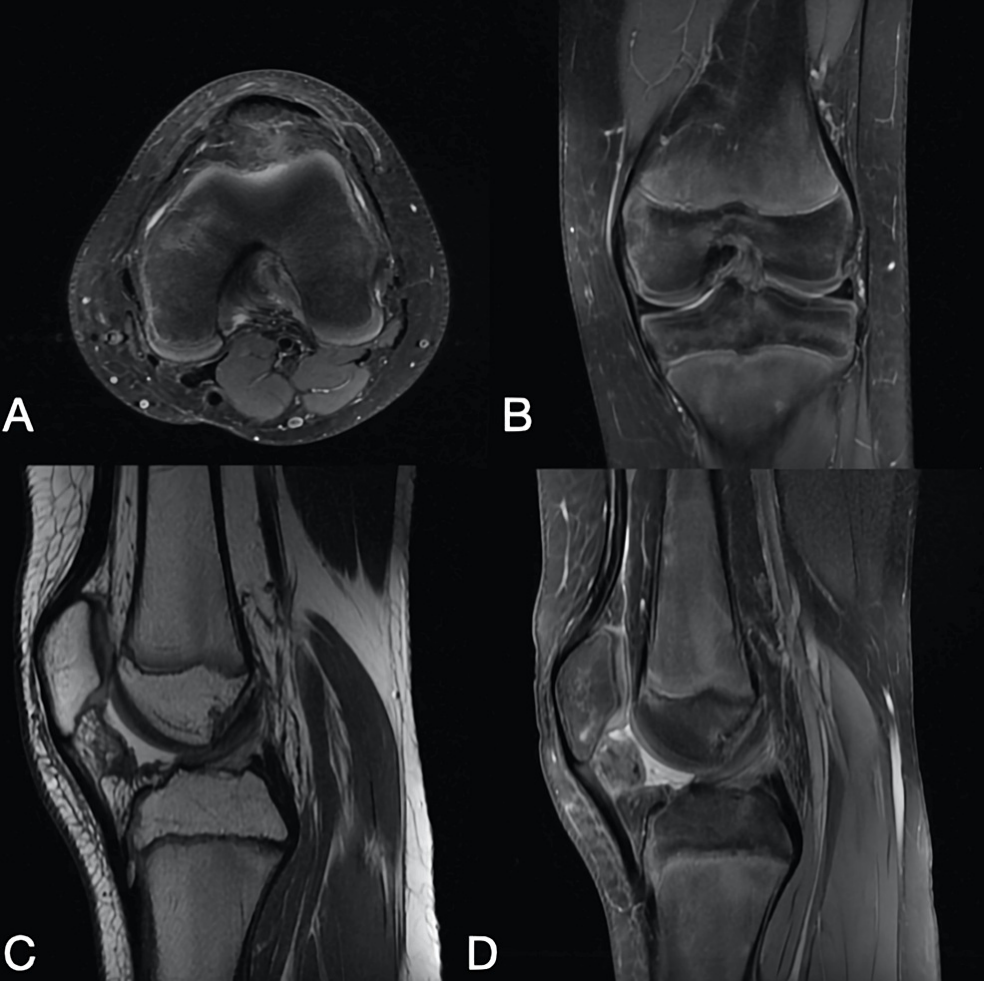
A: Four months postoperative left knee MRI in axial, B: Coronal, C and D: Sagittal planes shows a complete surgical removal of an ACL ganglion cyst.

Subsequently, arthroscopy of the left knee was performed under general anesthesia with the use of a tourniquet. During the exploration, a partially avulsed part of the anterior tibial spine (Figure 2) (in the insertion of ACL) of around 1 by 2 mm was observed and then excised. An embedded mass representing a ganglion-like structure was also seen within the ACL, which was drained to preserve the ACL. Before closure, the ACL was tested and found to be intact. The patient had an uneventful hospital stay with weight bearing as tolerated. The patient had three follow-up visits at two weeks, one month, and four months during which no pain was reported, and a full range of motion was observed. In addition, the patient went back to playing football with no complaints. Repeated MRI images (Figure 3) confirmed the absence of any abnormalities.

In conclusion, this case report describes a rare occurrence of a ganglion cyst in the anterior cruciate ligament that caused knee pain in a pediatric patient. The use of MRI and arthroscopy led to a proper diagnosis and successful treatment of the patient, resulting in a pain-free full range of motion.

## Discussion

Ganglion cysts are commonly found near joints, with the wrist being their preferred location. They are significantly less common in and around the knee, and when present, they are usually associated with the meniscus or joint capsule. Other possible locations include tendons, bones, muscles, and the infrapatellar fat pad [2]. The medical literature contains only seven case reports on the occurrence of cysts linked with the anterior cruciate ligaments (ACL) of the knee joint, with no case series reports [3,4]. Children in the case reports previously published ranged in age from "7 to 18 years, with an average age of 12.3 years and a gender distribution of four males and three females" [1,4,5]. During a routine autopsy in 1924, Caan documented the ACL ganglion cyst for the first time [6]. With the widespread use of MRI and arthroscopy seen in practice, an increasing number of instances are being reported [7,8]. Battaglia TC et al. reported the youngest age of a similar presentation, which was two years old [9]. Jawish et al. reported the case of a seven-year-old child in another study [3].

Although how or why ganglion cysts form is disputed, theories suggest that synovium may herniate into surrounding tissues during development or that pluripotent mesenchymal cells may experience degenerative and proliferative changes in response to trauma [10,11]. Cruciate ligament ganglion cysts are a common pathology that may result from various causes. Among them, trauma has been suggested as a prevalent etiology due to its capacity to elicit the release of hyaluronic acid, a mucinous substance. The hypothesis is that hyaluronic acid, upon being released, permeates the tissue planes, creating capsular channels that ultimately coalesce into a cystic shape. As the anterior cruciate ligament (ACL) is an extrasynovial structure, any irritation or trauma to the synovium that envelopes the ACL can trigger the liberation of hyaluronic acid, leading to the production of mucin and potentially promote the development of ganglion cysts. It is worth noting that the pathogenesis of cruciate ligament ganglion cysts is still not fully understood and that further research is required to elucidate the underlying mechanisms. Nonetheless, the association between trauma and the release of hyaluronic acid, as well as the subsequent formation of cystic ganglia, is a plausible explanation that merits consideration in the evaluation and management of this condition [12]. The fact that the cysts were mostly found in men (male: female ratio, 21:10) supports this conclusion, as females are assumed to be less likely to suffer trauma and sporting injuries, which meta-analyses have confirmed. The presence of cysts, mainly behind the ACL, showed that mechanical stress and microtrauma generated by repetitive knee motion could be the reason [13].

The clinical presentation of an intra-articular anterior cruciate ligament (ACL) cyst is believed to exhibit considerable heterogeneity. While many patients remain asymptomatic and are incidentally diagnosed on magnetic resonance imaging (MRI), symptomatic individuals typically complain of indeterminate knee pain that localizes to the medial joint line, lateral joint line, or retropatellar regions [3,4,14]. Upon physical examination, patients often exhibit an intact and functional ACL, with negative findings on the anterior drawer and Lachman tests, indicating that the cyst is not interfering with the mechanical stability of the knee [15]. Furthermore, patients may report other common symptoms such as joint swelling, popping sensations, effusion, clicking, a limited range of motion, and difficulty with full flexion and extension of the knee joint. It is essential to note that these clinical manifestations are nonspecific and may overlap with those of other knee pathologies. Consequently, a comprehensive assessment, including detailed history-taking, physical examination, and imaging studies, is necessary to establish a definitive diagnosis and provide optimal care for patients with intra-articular ACL cysts [1,4,14,15].

The present case report is unique in that it describes a ganglion cyst embedded in the tendon rather than in the root, as seen in previously reported cases. This finding suggests that intra-articular ganglia may have a wider range of presentations than previously thought. Another interesting observation is that the patient exhibited a 10-degree loss of extension, which resolved after arthroscopy. This underscores the importance of a thorough evaluation of the knee joint, as the loss of extension could have been missed if not carefully examined.

In a seminal study conducted by Krudwig et al., a total of 85 cases of intra-articular ganglion cysts were reported, with the majority of cases being asymptomatic (76 cases). However, nine patients presented with symptomatic cysts, and interestingly, none of these individuals had a history of trauma. The observation of a definite history of trauma in our patient with an intra-articular ganglion cyst is a noteworthy finding. While the pathogenesis of these cysts remains incompletely understood, several potential mechanisms have been proposed, including synovial herniation, capsular mucoid degeneration, and embryonic remnants. Trauma has also been suggested as a possible contributing factor, possibly causing microtrauma to the joint capsule or synovial membrane and leading to the formation of a cystic lesion. In light of this, the presence of a clear history of trauma in our patient raises the possibility of a causative relationship between the traumatic event and the development of the ganglion cyst. Further investigations, such as imaging studies and follow-up assessments, will be essential to gain a better understanding of the underlying mechanisms and guide appropriate management of this condition [14]. MRI has several advantages over other imaging modalities in diagnosing intra-articular ganglion cysts, including high sensitivity, specificity, and accuracy, as well as being non-invasive. MRI can provide detailed information about the cyst's size, location, and relationship with adjacent structures, as well as to rule out other concomitant pathologies that may mimic the clinical presentation. Additionally, MRI can help guide the appropriate management of intra-articular ganglion cysts, whether conservative or surgical, by providing information on the cyst's contents and the degree of intra-articular disruption. Therefore, when an intra-articular ganglion cyst is suspected, MRI should be considered the imaging modality of choice to confirm the diagnosis and guide treatment decisions. A comprehensive and accurate diagnosis is essential to optimize the management of and improve the clinical outcomes for patients with this rare but potentially debilitating pathology [1,16,17].

Brown and Dandy reported that arthroscopic removal is the gold standard treatment for individuals with symptomatic intra-articular ganglion cysts, with approximately 95% of patients reporting satisfactory outcomes [18]. Despite the need for hospitalization and general anesthesia, this technique has a low recurrence rate [18]. Computed tomography (CT) or ultrasound-guided percutaneous aspiration may be considered as potential alternatives. However, the benefits of these outpatient procedures, such as a shorter recovery period and less invasiveness, must be weighed against the significantly high risk of recurrence [19]. In our patient, complete arthroscopic drainage of the cyst was performed, resulting in a successful outcome with complete resolution of symptoms.

In summary, this case report underscores the importance of considering intra-articular ganglia as a possible cause of knee pain and highlights the need for a thorough evaluation of the knee joint. It also suggests that intra-articular ganglia may present in a more diverse manner than previously thought.

## Conclusions

Ganglion cysts of the cruciate ligament are uncommon conditions with vague symptoms. In situations involving internal knee derangements, the diagnosis of intra-articular ganglion cysts should be explored. This is especially crucial if a patient does not respond to initial treatment. Trauma can cause alterations in ectopic dormant synovial tissue, which can result in cyst development. Before the arthroscopic procedure, MR imaging is used to confirm the cyst lesions and provide useful information for preoperative planning. It also aids in the exclusion of any other related intra-articular lesions. The preferred treatment is arthroscopic resection. Our case illustrates a rare manifestation of an intra-articular cyst implanted in the ACL that was successfully treated with arthroscopic drainage, resulting in complete symptom relief.
